# Validation of the Arabic Version of the Chronic Heart Failure Health-Related Quality of Life Questionnaire in Jordan

**DOI:** 10.3390/healthcare14081076

**Published:** 2026-04-17

**Authors:** Walid Al-Qerem, Sawsan Khdair, Anan Jarab, Akram Saleh, Mohammad Al-Rawashdeh, Judith Eberhardt, Walaa Ashran, Lama Sawaftah, Fawaz Alasmari, Alaa Hammad, Nouf Alsultan

**Affiliations:** 1 Department of Pharmacy, Faculty of Pharmacy, Al-Zaytoonah University of Jordan, Amman 11733, Jordan; sawsan.khdair@zuj.edu.jo (S.K.); or 202227070@std-zuj.edu.jo (W.A.); lama.sawaftah@zuj.edu.jo (L.S.); alaa.hammad@zuj.edu.jo (A.H.); 2 Department of Clinical Pharmacy, Faculty of Pharmacy, Jordan University of Science and Technology, Irbid 22110, Jordan; asjarab@just.edu.jo; 3 Cardiology Department, Jordan University Hospital, Amman 11942, Jordan; a.saleh@ju.edu.jo (A.S.); dr.mohrawashdeh88@gmail.com (M.A.-R.); 4 Department of Psychology, School of Social Sciences, Humanities and Law, Teesside University, Borough Road, Middlesbrough TS1 3BX, UK; j.eberhardt@tees.ac.uk; 5 Department of Clinical Pharmacy, The University of Jordan Hospital, The University of Jordan, Amman 11942, Jordan; 6 Department of Pharmacology and Toxicology, College of Pharmacy, King Saud University, Riyadh 4545, Saudi Arabia; ffalasmari@ksu.edu.sa; 7 Faculty of Medicine and Health Sciences, Medical School, University of Nottingham, Nottingham NG7 2QL, UK; alxna75@nottingham.ac.uk

**Keywords:** heart failure, quality of life, Jordan, adults, validation

## Abstract

**Objectives**: We aimed to evaluate the reliability and validity of the Arabic version of the Chronic Heart Failure Health-Related Quality of Life Questionnaire (CHFQOLQ-20) among patients with heart failure in Jordan. **Methods**: A cross-sectional study was conducted among 399 adults with heart failure recruited from a tertiary hospital in Jordan (median age 68 years; 55.9% male). The CHFQOLQ-20 was translated using forward–backward procedures. Construct validity was examined using confirmatory factor analysis (CFA) and a multidimensional Partial Credit Model. Differential item functioning by sex and internal consistency were assessed. **Results**: CFA supported the original four-domain structure (physical, cognitive, mental, and general health), with all items showing significant factor loadings. Item-level analyses demonstrated acceptable model fit, ordered response thresholds, and minimal sex-related bias. Physical health scores were lower than other domains. **Conclusions**: The Arabic CHFQOLQ-20 is a valid, reliable, and multidimensional measure of HRQoL in patients with heart failure, supporting its use in clinical practice and research.

## 1. Introduction

Heart failure (HF) affects an estimated 64 million people worldwide and is associated with high levels of morbidity and mortality [1,2]. In Jordan, HF is a common cardiovascular condition and a major contributor to hospital admissions [3], with reported in-hospital mortality of 9.6% [4] and a 30-day mortality rate of 9.5% [5]. Compared with healthy individuals and those living with other chronic conditions, patients with HF experience substantially poorer quality of life (QoL). This reduction is driven by the physical and psychological burden of the disease, including dyspnea, fluid retention, chest pain, sleep disturbances, generalized weakness, depression, and anxiety, all of which impair physical functioning and disrupt social participation, ultimately compromising health-related quality of life (HRQoL) [6].

Evidence from Jordan reflects this burden. A cross-sectional study found that 62% and 65% of patients with HF reported clinically significant anxiety and depression, respectively, based on the Hospital Anxiety and Depression Scale (HADS), alongside poor QoL as measured by the 36-Item Short Form Health Survey (SF-36) [7]. HRQoL is also closely linked to disease severity, as reflected by the New York Heart Association (NYHA) classification, with poorer QoL consistently associated with worse prognosis [8,9,10]. Importantly, a large multicenter cohort study demonstrated that QoL is a major and independent predictor of both hospital admission and all-cause mortality among patients with HF [11]. Together, these findings highlight the importance of routine and systematic assessment of QoL in individuals with chronic HF, not only to inform care but also to support improved clinical outcomes and survival [12].

Given the inherent complexity of quality of life (QoL), numerous instruments have been developed to capture its multidimensional and subjective nature [13]. Conceptually, the present study is grounded in the Wilson–Cleary model of health-related quality of life, later refined by Ferrans and colleagues, which links biological and symptom status to functional status, general health perceptions, and overall quality of life [14,15]. Within this framework, the physical and mental domains reflect the symptomatic and emotional consequences of heart failure, whereas the general health domain captures the patient’s global appraisal of health. Although a range of tools has been used to assess QoL in patients with heart failure (HF), each has notable limitations. Generic measures such as the 36-Item Short Form Health Survey (SF-36) and the Nottingham Health Profile (NHP) lack disease specificity, include items that are not directly relevant to HF, and show limited sensitivity to small but clinically meaningful changes in symptoms [16]. Disease-specific instruments, including the Minnesota Living with Heart Failure Questionnaire (MLHFQ) [17], the Kansas City Cardiomyopathy Questionnaire (KCCQ) [18], the Quality of Life in Severe Heart Failure Questionnaire (QLQ-SHF) [19], and the Chronic Heart Failure Questionnaire (CHFQ) [20] address some of these limitations but are not without shortcomings [21]. Both the KCCQ and MLHFQ may underestimate the negative impact of HF-related complications, potentially leading to an overestimation of improvements in QoL [22]. The CHFQ, meanwhile, was developed using a relatively small sample and has been criticized for being burdensome to administer in routine settings [20,23]. Importantly, despite strong evidence linking cognitive dysfunction to poorer outcomes in HF, including increased hospitalization and mortality, most existing QoL instruments do not adequately capture cognitive functioning. In this conceptual model, cognition is best understood as a patient-relevant aspect of functional status because difficulties with memory, attention, and decision-making affect self-care, treatment adherence, symptom interpretation, and the way illness burden is translated into everyday life. With the exception of a single item in the MLHFQ, cognitive health is largely absent from currently available questionnaires [24].

To address these limitations, the Chronic Heart Failure Health-Related Quality of Life Questionnaire (CHFQOLQ-20) was developed as a concise and user-friendly instrument designed specifically for patients with heart failure. The questionnaire comprises 20 items covering four key domains of health-related quality of life (HRQoL), physical, cognitive, mental, and general health, thereby capturing aspects of functioning that are often underrepresented or absent in existing HF-specific measures [24]. In particular, the explicit inclusion of a cognitive domain represents an important advance, given the well-documented impact of cognitive impairment on disease management, health outcomes, and everyday functioning in patients with HF. Rather than functioning independently of the other domains, this cognitive component is expected to interact with physical symptoms, emotional well-being, and general health perceptions, thereby offering a more coherent representation of HRQoL in HF [14,15,25,26,27].

Despite its promising psychometric properties in the original development study, the applicability of the CHFQOLQ-20 to Arabic-speaking populations has not yet been established. To date, no study has used confirmatory factor analysis to examine whether the proposed four-domain structure of the CHFQOLQ-20 holds in an Arabic-speaking HF population. Establishing cross-cultural validity is essential before the instrument can be confidently used in clinical or research settings. Accordingly, the present study aimed to evaluate the reliability and construct validity of the Arabic version of the CHFQOLQ-20 among Jordanian patients with heart failure.

## 2. Materials and Methods

### 2.1. Study Design and Participants

This cross-sectional study was conducted between August and December 2025 at Jordan University Hospital in Amman, Jordan. A total of 399 patients with heart failure (HF) were recruited during routine outpatient cardiology clinic visits and inpatient admissions. Eligible participants were adults aged 18 years or older who had a confirmed diagnosis of HF, were Arabic-speaking, able to communicate effectively, and willing to participate. When appropriate, caregivers assisted with participation.

All participants were approached by a trained researcher and provided with a clear explanation of the study aims and procedures prior to enrolment. Written informed consent was obtained from all participants, and participation was entirely voluntary. Confidentiality of all collected data was strictly maintained throughout the study. Ethical approval was granted by the Institutional Review Board of Jordan University Hospital (Reference No: 10/2025/21196; dated 19 May 2025), and the study was conducted in accordance with the principles of the Declaration of Helsinki.

### 2.2. Study Instrument

Sociodemographic and clinical data were collected using a structured form developed by the research team. Information included participants’ age, sex, marital status, educational level, monthly income, smoking status, duration of heart failure, and the number of prescribed medications for heart failure management. Clinical information was verified through review of patients’ medical records.

Health-related quality of life (HRQoL) was assessed using the 20-item Chronic Heart Failure Health-Related Quality of Life Questionnaire (CHFQOLQ-20) [23]. The CHFQOLQ-20 comprises four domains: physical health (8 items), cognitive health (6 items), mental health (3 items), and general health (3 items). Items are rated on a 5-point Likert scale ranging from “very much” to “not at all,” with higher scores indicating better HRQoL.

### 2.3. Tool Validation

The translation and cultural adaptation of the CHFQOLQ-20 into Arabic followed a standardized forward–backward translation procedure based on Brislin’s methodology and established cross-cultural adaptation guidance [28,29]. The original English version was independently translated into Arabic by two bilingual translators whose native language was Arabic. The translators worked separately, and no additional blinding procedures beyond separation of translation stages were implemented. The two forward translations were then compared item by item in a consensus meeting involving the translators and the research team, and discrepancies were resolved by prioritizing conceptual equivalence, clarity, and natural Arabic wording rather than literal translation. This version was then independently back-translated into English by two additional bilingual translators who were not involved in the forward-translation stage. The back-translated versions were compared with the original instrument to identify inconsistencies, ambiguities, or conceptual discrepancies. Following review and refinement by the research team, a final Arabic version was produced, ensuring semantic and conceptual equivalence with the original questionnaire.

Content validity of the Arabic CHFQOLQ-20 was evaluated by an expert panel comprising two cardiologists and a clinical pharmacist. Panel members independently assessed item relevance, clarity, comprehensiveness, and cultural appropriateness for Jordanian patients with HF. Comments were discussed with the research team and resolved by consensus, and the panel confirmed that the translated items adequately represented the quality-of-life construct in heart failure.

Face validity was assessed through pilot testing with a sample of 30 patients with heart failure who met the study inclusion criteria. Participants completed the questionnaire and provided feedback on item clarity, wording, and ease of comprehension. In addition, brief cognitive-debriefing probes were used to explore how participants interpreted selected items and response options and whether any wording felt ambiguous or culturally unfamiliar [29]. Minor linguistic adjustments were made in response to this feedback. Data from the pilot phase were not included in the final analyses.

Construct validity was examined using confirmatory factor analysis (CFA) to test the proposed four-factor structure and to evaluate the relationships between observed items and their corresponding latent domains. To further assess item-level performance, a partial credit model (PCM) was applied. Item fit was evaluated using infit and outfit mean square statistics, with most items demonstrating acceptable model fit. Threshold (Thurstone) parameters were examined to assess the ordering of response categories and were generally found to be appropriately ordered.

### 2.4. Statistical Analysis

Before conducting the analyses, the data were examined for completeness, distributional characteristics, and the suitability of handling item responses as ordinal variables. Descriptive statistics for continuous variables were reported as means with standard deviations or medians with interquartile ranges, depending on data distribution, whereas categorical variables were summarized using counts and percentages.

Because the questionnaire items were ordinal, confirmatory factor analysis (CFA) was performed using estimation methods appropriate for ordered data to evaluate the proposed four-factor model (Cognitive, General, Mental, and Physical). Model fit was assessed using the Comparative Fit Index (CFI), Tucker–Lewis Index (TLI), and Standardized Root Mean Square Residual (SRMR). Item adequacy was evaluated through standardized factor loadings and their associated z values, all of which were statistically significant (*p* < 0.001).

Internal consistency reliability was examined for the overall scale and each subscale using ordinal reliability coefficients. All statistical analyses were carried out using R software (version 4.5.2).

## 3. Results

A total of 399 patients with heart failure were included in the study. As shown in Table 1, the median age of participants was 68 years (IQR: 60–77). Most participants were male (55.9%), had completed secondary education (30.1%), were married (63.7%), reported a monthly income of less than 500 JOD (53.6%), and were non-smokers (42.9%). The median duration of heart failure was 3 years (IQR: 1.5–7), and the median number of medications prescribed for heart failure management was four. Full sociodemographic and clinical characteristics of the study sample are presented in Table 1.

Descriptive statistics for the four CHFQOLQ-20 domains are presented in Table 2. The physical domain, comprising eight items with a possible sum score ranging from 0 to 32, showed the lowest per-item mean (M = 1.69, SD = 1.22) and a median of 1.75 (IQR = 2.12). Mean scores for individual physical items ranged from 1.14 to 2.48.

The cognitive domain consisted of six items (possible range 0–24) and demonstrated higher average scores, with a mean per-item score of 2.67 (SD = 0.93) and a median of 2.83 (IQR = 1.17). Item-level means in this domain ranged from 1.91 to 3.26. For the general domain, which included three items (possible range 0–12), the mean per-item score was 2.57 (SD = 1.45), with a median of 3.00 (IQR = 2.67). Item means were relatively consistent, ranging from 2.39 to 2.67. The mental domain, also comprising three items with a possible range of 0–12, showed the highest per-item mean (M = 2.73, SD = 1.32) and a median of 3.00 (IQR = 2.00), with item means ranging from 2.56 to 2.83. Across all domains, responses spanned the full 0–4 range at the item level, and observed total scores covered the entire possible range for each domain. Item-level response distributions for the CHFQOLQ-20 are provided in Table A1 (see Appendix A).

Confirmatory factor analysis (CFA) was conducted to evaluate the four-factor structure proposed in the original CHFQOLQ-20. The results supported the adequacy of the model, with all 20 items loading appropriately onto their respective domains. Standardized factor loadings, along with standard errors, z values, and *p* values for each item, are presented in Table 3. CFA showed good model fit χ^2^(160) = 978.82, *p* < 0.001, CFI = 0.999, TLI = 0.999, SRMR = 0.072.

Factor loadings were generally high across domains. The strongest loadings were observed for item 15 (1.00) and item 17 (0.999), while the lowest loading was observed for item 10 (0.774). All standardized loadings were statistically significant, indicating that each item contributed meaningfully to its intended latent construct. The very high loadings observed for several items, particularly within the physical, mental, and general domains, indicate strong within-domain homogeneity but may also suggest some degree of item redundancy or local dependence.

A multidimensional Partial Credit Model (PCM) was applied to examine item functioning across the cognitive, general, mental, and physical domains of quality of life. The likelihood ratio test was statistically significant, supporting the suitability of the multidimensional PCM for the present data.

Item fit statistics are presented in Table 4. Overall, most items demonstrated acceptable model fit, with infit and outfit mean square (MNSQ) values generally within recommended ranges. Items in the cognitive domain showed particularly stable fit, with infit and outfit values close to unity. Within the physical domain, items QoL_7 and QoL_8 exhibited relatively higher infit values, indicating greater response variability; however, these values remained within acceptable limits. This pattern suggests that activity-restriction items may capture a broader and more heterogeneous range of physical limitations than the other domains, even though their measurement performance remained acceptable.

Examination of threshold parameters indicated that response categories were appropriately ordered across all items (Cat1 < Cat2 < Cat3 < Cat4), supporting the use of the four-category response format. Threshold estimates spanned a wide range of the latent trait, suggesting that the items were able to capture varying levels of quality-of-life impact across domains.

Taken together, the item fit statistics and ordered thresholds confirm the adequacy of the item hierarchy and support the ability of the scale to differentiate between respondents with differing levels of quality-of-life impairment.

**Figure 1:**
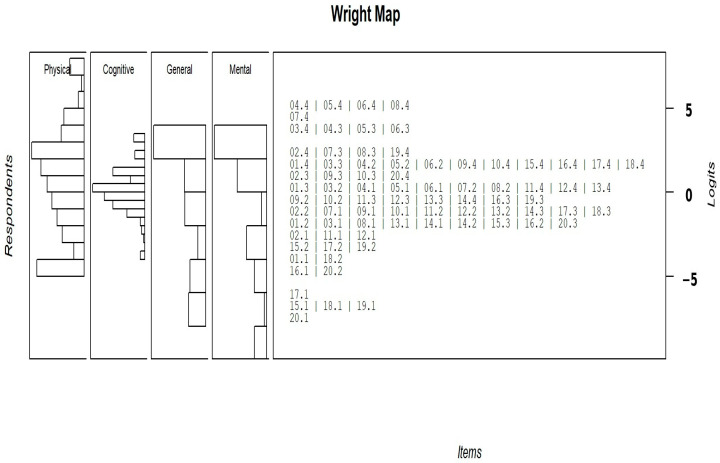
Wright map for the CHFQOLQ-20.

The Wright map (Figure 1) illustrates the alignment between participants’ quality-of-life levels and the distribution of CHFQOLQ-20 item thresholds along the latent trait continuum. The person distribution shows coverage across a broad range of quality-of-life levels, with most respondents concentrated around the center of the scale. Item thresholds were well dispersed across the continuum, indicating appropriate targeting of items to the sample. Overall, these findings support the scale’s capacity to differentiate between respondents across the intended measurement range.

**Figure 2:**
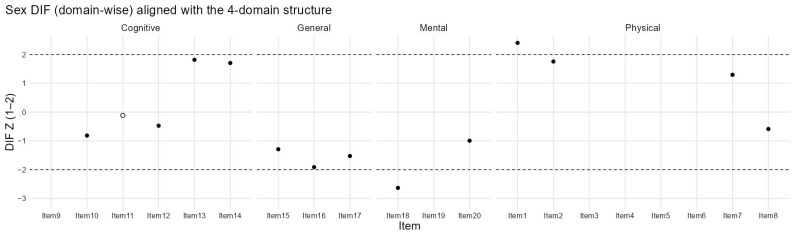
Sex-based DIF across CHFQOLQ-20 domains. The white circle is the reference group.

**Figure 3:**
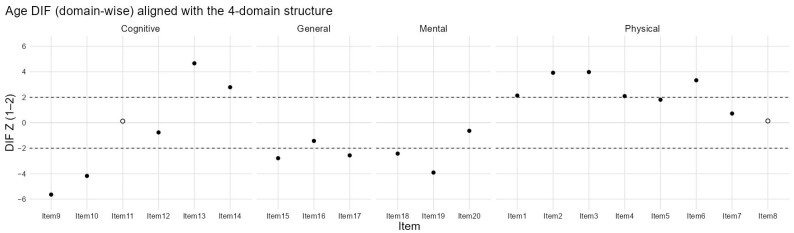
Age-based DIF across the four questionnaire domains using a median split at 68 years. The white circle is the reference group.

Figure 2 presents the sex-based differential item functioning (DIF) analysis across the four domains of the CHFQOLQ-20. Most items within the cognitive, general, mental, and physical domains showed values within the acceptable range (±2), indicating minimal evidence of sex-related item bias. A small number of items in the physical domain approached or marginally exceeded this threshold, suggesting minor sex-related differences in item endorsement; however, these effects were limited in magnitude and showed no consistent directional pattern. Overall, the results support measurement invariance of the CHFQOLQ-20 across sex. Additional exploratory DIF analysis was conducted using the same multifacet partial credit model for age. Age was dichotomized at the sample median of 68 years (<68 years, n = 194; >=68 years, n = 205). The age-based DIF analysis showed a broader pattern of differential functioning than that observed for sex, with several items exceeding the conventional absolute z threshold of 2. The most prominent deviations were observed for items 9, 10, 13, and 14 within the cognitive domain and for items 1, 2, 3, 4, and 6 within the physical domain, while smaller numbers of flagged items were also observed in the general and mental domains (Figure 3).

## 4. Discussion

This study evaluated the reliability and validity of the Arabic version of the Chronic Heart Failure Health-Related Quality of Life Questionnaire (CHFQOLQ-20) in Jordanian adults with heart failure. Given the physical, psychological, and social burden associated with heart failure, having a valid and culturally appropriate tool to assess quality of life is important for both clinical care and research. To date, the psychometric properties of the CHFQOLQ-20 had not been examined in Arabic-speaking populations, and this study addresses that gap. This study builds on the original development of the CHFQOLQ-20 by testing its performance in a large sample of patients with heart failure in Jordan, providing evidence for its use in a different linguistic and cultural context. This approach is consistent with recommendations to re-evaluate patient-reported outcome measures when they are adapted for use across cultures and languages [28,30].

Confirmatory factor analysis supported the four-factor structure of the CHFQOLQ-20, encompassing physical, cognitive, mental, and general health domains. All items demonstrated statistically significant standardized loadings on their respective factors, indicating that the translated items function as intended and capture the constructs they were designed to measure. Importantly, these findings suggest that the conceptual organization of the questionnaire is preserved in the Arabic version, rather than being altered by linguistic or cultural adaptation. Several loadings were also extremely high, especially in the physical, mental, and general domains. While this pattern supports strong internal coherence, it may indicate that some items within the shorter subscales are very closely related in content and could share variance beyond the intended latent construct.

Support for this four-domain structure reinforces the view that quality of life in heart failure is inherently multidimensional and cannot be adequately captured by measures focused solely on physical symptoms or emotional well-being. The alignment of physical, psychological, cognitive, and general health domains reflects the interconnected ways in which heart failure affects daily functioning and overall health. This pattern is also consistent with the Wilson–Cleary/Ferrans conceptual model, in which symptoms and functional consequences shape broader health perceptions and overall quality of life [14,15]. Similar multidimensional structures have been reported in other heart failure-specific quality-of-life instruments [31,32], lending further support to the construct validity of the Arabic CHFQOLQ-20 and its suitability for use in both clinical and research settings.

In the present study, the cognitive domain demonstrated strong psychometric performance. Cognitive impairment is a common feature of many chronic conditions, including heart failure, and has important implications for both patients and health care systems. A systematic review of 22 controlled studies examining the relationship between heart failure and cognitive decline identified a pattern of generalized cognitive dysfunction, particularly affecting memory, attention, mental flexibility, and overall cognitive functioning [27]. Cognitive impairment in heart failure has also been linked to poorer medication adherence, higher rates of hospitalization, and worse clinical outcomes [25,26]. Despite this evidence, cognitive functioning is often underrepresented in commonly used quality-of-life instruments for heart failure. The strong factor loadings and stable item performance observed for the cognitive domain in this study suggest that the CHFQOLQ-20 captures this aspect of patients’ experience in a reliable and meaningful way. Conceptually, this domain appears to bridge physical and emotional burden with broader health appraisal, because difficulties in attention, memory, and decision-making can intensify problems in symptom interpretation and everyday self-management [25,26,27]. More specifically, deficits in memory, attention, and executive functioning may reduce patients’ ability to follow complex medication regimens, recognize symptom worsening, adhere to dietary and fluid recommendations, and seek timely care, thereby directly influencing disease management [25,26]. From a clinical standpoint, lower scores in this domain may help identify patients who need simplified education, repeated counseling, medication-management support, caregiver involvement, or closer follow-up, because cognitive difficulties can compromise symptom monitoring and self-care behaviors that are central to heart failure management [25,26]. These findings support the inclusion of cognitive functioning as a core component of quality-of-life assessment in heart failure, particularly given its close links to disease management and outcomes.

Item-level analysis using the Partial Credit Model further supported the robustness of the Arabic CHFQOLQ-20. Most items showed acceptable infit and outfit statistics, indicating appropriate item functioning and consistency with model expectations. Items within the cognitive and mental domains demonstrated particularly stable fit, suggesting that these items were interpreted consistently by respondents. Some items in the physical domain showed greater response variability; however, these values remained within acceptable limits. Rather than indicating weaknesses in the instrument, this variability likely reflects the heterogeneous symptom profiles and functional limitations experienced by patients with heart failure [33]. At the same time, the higher infit values for QoL_7 and QoL_8 indicate that activity restriction and participation-related items may be more context dependent than other physical items, potentially reflecting differences in comorbidity burden, mobility, or day-to-day role expectations.

The presence of ordered response thresholds across all items indicates that participants were able to distinguish meaningfully between the response options. This finding supports the appropriateness of the Likert-type response format used in the CHFQOLQ-20 and suggests that the scale can capture gradations in quality-of-life impairment. From an item response theory perspective, well-ordered thresholds are a key indicator of effective category functioning and overall scale performance [34,35].

The Wright map analysis further supports the adequacy of the CHFQOLQ-20 by demonstrating good targeting between item thresholds and participants’ quality-of-life levels. Item thresholds covered a broad span of the latent trait and aligned well with the distribution of respondents, indicating that the questionnaire is able to distinguish between different degrees of quality-of-life impairment in patients with heart failure. Although small gaps were observed at the extremes of the continuum, this pattern is commonly seen in health-related quality-of-life measures and is unlikely to meaningfully affect overall measurement precision.

Findings from the sex-based differential item functioning analysis further strengthen confidence in the instrument. Most items showed DIF values within acceptable limits, indicating minimal sex-related bias. While a small number of physical domain items demonstrated marginal DIF, these effects were limited in size and showed no consistent directional pattern. Taken together, these results suggest that the Arabic version of the CHFQOLQ-20 functions similarly for men and women and can be used to assess quality of life in mixed-gender heart failure populations.

The more pronounced DIF pattern observed across age groups is clinically plausible in heart failure [36,37,38,39,40]. Older and younger patients may differ in functional reserve, symptom appraisal, adaptation to chronic disease, and the way they interpret cognitive and general-health items, even when their overall health-related quality of life burden is comparable [36,37,38,39,40]. For that reason, the age-related DIF observed here may reflect meaningful heterogeneity in lived experience rather than simple item bias alone [41,42]. At the same time, these analyses were exploratory and based on a median-split comparison, so they should not be taken as definitive evidence against the use of the scale across age groups [41,42,43]. Future studies should examine invariance using prespecified age strata and larger multicenter samples, and should assess whether the observed DIF materially affects domain-level or total-score comparisons between younger and older patients [41,42,44].

In this study, scores in the physical health domain were lower than those observed in the cognitive, mental, and general health domains. This pattern is expected, as physical symptoms such as fatigue and dyspnea are core features of heart failure and are known to substantially limit daily activities and independence [45,46]. The lower physical health scores observed here are consistent with previous findings in the literature. For example, a cross-sectional study from Saudi Arabia reported moderate to severe limitations in basic physical activities among patients with heart failure [47]. Similarly, research conducted in Jordan identified a high symptom burden that was strongly associated with poorer physical quality of life and greater restrictions in everyday functioning [48]. Together, these findings highlight the substantial impact of symptom burden on physical health and highlight the importance of systematically assessing and addressing physical limitations as part of comprehensive care aimed at improving overall well-being in patients with heart failure.

### Strengths, Limitations and Future Directions

This study has several strengths. The relatively large sample size enhances confidence in the stability of the findings, while the combined use of confirmatory factor analysis and item response theory modeling allowed for a detailed evaluation of both the underlying factor structure and individual item performance. In addition, to our knowledge, this is the first study to validate the CHFQOLQ-20 in an Arabic-speaking population with heart failure, addressing an important gap in the literature.

Some limitations should also be considered. First, the cross-sectional design precluded assessment of test–retest reliability and responsiveness to clinical change over time. Future longitudinal studies are needed to evaluate the temporal stability of the Arabic CHFQOLQ-20 and its sensitivity to treatment effects or disease progression. Second, although the translation process included expert review and pilot debriefing, a more extensive qualitative validation phase with in-depth cognitive interviewing was not undertaken. Third, participants were recruited from a single tertiary care hospital, which may limit the generalizability of the findings. Although the hospital serves patients from across Jordan, future research should validate the instrument across multiple clinical contexts, including acute inpatient care, outpatient follow-up, cardiac rehabilitation, community-based management, and primary care, to capture a broader spectrum of disease severity, care pathways, health care access, and self-management demands. Further validation in other Arabic-speaking countries would also be valuable, as it would allow examination of the questionnaire’s performance across different cultural, socioeconomic, and health care system contexts, strengthening its applicability for regional and cross-national research.

## 5. Conclusions

This provides evidence supporting the validity and reliability of the Arabic version of the CHFQOLQ-20 for assessing health-related quality of life in Jordanian patients with heart failure. The confirmation of its four-domain structure, together with stable item functioning and appropriate targeting across quality-of-life levels, supports the use of the questionnaire as a multidimensional measure that reflects the lived experience of heart failure.

Importantly, the inclusion of a cognitive domain addresses a well-recognized but often overlooked aspect of quality of life in this population. As such, the Arabic CHFQOLQ-20 offers a practical and conceptually sound tool that can be used in both clinical settings and research to support more comprehensive assessment, monitoring, and evaluation of outcomes in patients with heart failure.

## Figures and Tables

**Table 1:** Participants’ sociodemographic characteristics.

		Count (%), Median (IQR)
Age		68 (60–77)
Gender	Female	176 (44.1%)
	Male	223 (55.9%)
Education level	Illiterate	64 (16%)
	Primary	81 (20.3%)
	Secondary	120 (30.1%)
	Diploma	38 (9.5%)
	Bachelor’s degree/Postgraduate studies	96 (24.1%)
marital status	not married	145 (36.3%)
	Married	254 (63.7%)
Average monthly income (JOD)	<500	214 (53.6%)
	500–1000	141 (35.3%)
	>1000	44 (11%)
Smoking status	No	171 (42.9%)
	Former smoker	121 (30.3%)
	Yes	107 (26.8%)
Duration of illness (years)		3 (1.5–7)
Number of medications used for congestive heart failure	1	6 (1.5%)
	2	16 (4%)
	3	53 (13.3%)
	4	161 (40.4%)
	5	126 (31.6%)
	6	33 (8.3%)
	7	4 (1%)

**Table 2:** Domain-Level Descriptive Statistics for the CHFQOLQ-20.

Domain	Items (k)	Mean per Item (SD)	Median per Item (IQR)	Range of Item Means	Observed Mean Range
Physical	8	1.69 (1.22)	1.75 (2.12)	1.14–2.48	0.00–4.00
Cognitive	6	2.67 (0.93)	2.83 (1.17)	1.91–3.26	0.00–4.00
General	3	2.57 (1.45)	3.00 (2.67)	2.39–2.67	0.00–4.00
Mental	3	2.73 (1.32)	3.00 (2.00)	2.56–2.83	0.00–4.00

**Table 3:** Standardized factor loadings (ordinal CFA model).

Factor	Item	Loading	SE	z	*p*
Cognitive	QoL_14	0.916	0.015	60.75	<0.001
Cognitive	QoL_9	0.909	0.026	35.00	<0.001
Cognitive	QoL_13	0.887	0.016	54.75	<0.001
Cognitive	QoL_11	0.857	0.017	49.38	<0.001
Cognitive	QoL_12	0.854	0.017	48.99	<0.001
Cognitive	QoL_10	0.774	0.030	25.38	<0.001
General	QoL_15	1.000	0.002	593.95	<0.001
General	QoL_17	0.999	0.002	637.49	<0.001
General	QoL_16	0.925	0.009	98.98	<0.001
Mental	QoL_20	0.993	0.003	327.91	<0.001
Mental	QoL_18	0.992	0.003	326.23	<0.001
Mental	QoL_19	0.927	0.009	108.07	<0.001
Physical	QoL_2	0.991	0.003	347.06	<0.001
Physical	QoL_1	0.979	0.005	203.41	<0.001
Physical	QoL_5	0.977	0.004	261.61	<0.001
Physical	QoL_4	0.966	0.005	195.77	<0.001
Physical	QoL_3	0.960	0.005	191.00	<0.001
Physical	QoL_6	0.954	0.006	155.63	<0.001
Physical	QoL_8	0.874	0.014	63.07	<0.001
Physical	QoL_7	0.861	0.015	57.11	<0.001

**Table 4:** Item fit statistics (infit/outfit) and Thurstone thresholds (multidimensional PCM).

Factor	Item	Infit (MNSQ)	Outfit (MNSQ)	Infit (t)	Outfit (t)	Thurstone Cat1	Thurstone Cat2	Thurstone Cat3	Thurstone Cat4
Cognitive	QoL_9	1.00	0.97	−0.05	−0.43	−1.05	−0.41	0.69	1.98
Cognitive	QoL_10	1.17	1.34	2.40	4.13	−1.07	−0.38	0.74	1.69
Cognitive	QoL_11	0.97	1.02	−0.36	0.27	−2.34	−1.41	−0.48	0.16
Cognitive	QoL_12	0.99	1.01	−0.05	0.13	−2.23	−1.41	−0.42	0.19
Cognitive	QoL_13	0.86	0.80	−1.81	−1.76	−1.93	−1.28	−0.60	−0.02
Cognitive	QoL_14 *	0.80	0.71	−2.31	−2.45	−2.06	−1.65	−1.05	−0.33
General	QoL_15	0.74	0.46	−3.11	13.81	−6.54	−3.38	−1.55	1.68
General	QoL_16	1.51	1.03	4.74	12.44	−4.45	−1.87	−0.13	1.98
General	QoL_17	0.80	0.51	−2.30	12.54	−6.25	−3.34	−1.48	1.87
Mental	QoL_18	0.82	0.59	−2.15	15.81	−7.06	−4.08	−1.45	1.38
Mental	QoL_19	1.31	1.04	3.32	8.94	−6.81	−3.00	−0.28	2.53
Mental	QoL_20	0.76	0.51	−2.97	16.85	−7.41	−4.40	−1.84	1.24
Physical	QoL_1	0.72	0.63	−3.87	−0.70	−3.69	−1.68	0.21	1.92
Physical	QoL_2	0.68	0.58	−4.58	−0.80	−2.41	−0.84	0.89	2.68
Physical	QoL_3	0.82	0.71	−2.38	−0.42	−2.00	−0.07	1.44	3.50
Physical	QoL_4	0.77	0.59	−2.96	0.15	0.13	1.99	3.81	5.31
Physical	QoL_5	0.77	0.57	−3.05	0.16	0.11	1.64	3.55	5.37
Physical	QoL_6	0.86	0.63	−1.68	0.32	0.29	1.58	3.69	5.51
Physical	QoL_7	1.70	1.63	7.40	0.94	−1.27	0.06	2.30	4.37
Physical	QoL_8	1.58	1.38	6.45	0.77	−1.73	0.10	2.51	4.87

Note. Thurstone thresholds are expected to be ordered (Cat1 < Cat2 < Cat3 < Cat4). Gap diagnostics reflect separation between adjacent thresholds; * flagged items indicate gaps smaller than 0.50.

## Data Availability

The data supporting the findings of this study are available from the authors upon reasonable request. The data are not publicly available due to privacy and ethical restrictions.

## Abbreviations

The following abbreviations are used in this manuscript:

CFA	confirmatory factor analysis
CHFQOLQ-20	Chronic Heart Failure Health-Related Quality of Life Questionnaire
DIF	Differential item functioning
HRQoL	Health-related quality of life
PCM	Partial credit model
TLI	Tucker–Lewis Index
RMSEA	Root Mean Square Error of Approximation

## Appendix A

**Table A1:** Participants Responses to CHFQOLQ-20.

	Very Much	A Lot	Moderately	Very Little	Not at All
Have you had difficulty walking inside the house?	58 (14.5%)	51 (12.8%)	70 (17.5%)	83 (20.8%)	137 (34.3%)
Have you had difficulty walking a short distance of more than one street or 100 m?	86 (21.6%)	51 (12.8%)	73 (18.3%)	85 (21.3%)	104 (26.1%)
Have you had difficulty performing household tasks such as gardening, moving objects, or using a vacuum cleaner and carrying daily groceries?	98 (24.6%)	68 (17%)	71 (17.8%)	88 (22.1%)	74 (18.5%)
Have you had difficulty climbing one flight of stairs without stopping?	173 (43.4%)	89 (22.3%)	74 (18.5%)	34 (8.5%)	29 (7.3%)
Have you had difficulty walking fast for a distance of more than 100 m?	171 (42.9%)	75 (18.8%)	81 (20.3%)	43 (10.8%)	29 (7.3%)
Have you had difficulty lifting and carrying heavy objects such as furniture and luggage?	178 (44.6%)	66 (16.5%)	87 (21.8%)	41 (10.3%)	27 (6.8%)
Have you had to remain lying down or sitting during the day?	119 (29.8%)	55 (13.8%)	102 (25.6%)	74 (18.5%)	49 (12.3%)
Have you had difficulty exercising, leisure activities, and traveling?	106 (26.6%)	70 (17.5%)	109 (27.3%)	75 (18.8%)	39 (9.8%)
Have you felt tired and had low energy?	79 (19.8%)	60 (15%)	123 (30.8%)	91 (22.8%)	46 (11.5%)
Have you felt shortness of breath while performing physical activities and tasks?	78 (19.5%)	64 (16%)	126 (31.6%)	74 (18.5%)	57 (14.3%)
Have you had difficulty concentrating? (such as concentrating while reading or watching television)	19 (4.8%)	34 (8.5%)	80 (20.1%)	78 (19.5%)	188 (47.1%)
Have you had difficulty remembering things related to the past few days? (such as remembering where things are or appointment dates)	21 (5.3%)	32 (8%)	86 (21.6%)	75 (18.8%)	185 (46.4%)
Have you had difficulty learning new things?	27 (6.8%)	29 (7.3%)	63 (15.8%)	73 (18.3%)	207 (51.9%)
Have you had difficulty making decisions?	20 (5%)	14 (3.5%)	45 (11.3%)	83 (20.8%)	237 (59.4%)
How would you rate your general health currently?	51 (12.8%)	50 (12.5%)	48 (12%)	81 (20.3%)	169 (42.4%)
How would you rate your health currently compared to last year?	80 (20.1%)	58 (14.5%)	48 (12%)	52 (13%)	161 (40.4%)
How would you rate your quality of life currently?	54 (13.5%)	48 (12%)	49 (12.3%)	83 (20.8%)	165 (41.4%)
Have you felt that you are a burden on others?	41 (10.3%)	41 (10.3%)	61 (15.3%)	79 (19.8%)	177 (44.4%)
Have you felt sadness and depression?	44 (11%)	60 (15%)	71 (17.8%)	75 (18.8%)	149 (37.3%)
Have you felt useless or worthless?	38 (9.5%)	38 (9.5%)	57 (14.3%)	85 (21.3%)	181 (45.4%)

## References

[1] Shahim B., Kapelios C.J., Savarese G., Lund L.H. (2023). Global Public Health Burden of Heart Failure: An Updated Review. Card. Fail. Rev..

[2] Vollset S.E., Ababneh H.S., Abate Y.H., Abbafati C., Abbasgholizadeh R., Abbasian M., Abbastabar H., Al Magied A.H.A.A., ElHafeez S.A., Abdelkader A. (2024). Burden of disease scenarios for 204 countries and territories, 2022–2050: A forecasting analysis for the Global Burden of Disease Study 2021. Lancet.

[3] Saifan A.R., Abu Hayeah H., Ibrahim A.M., Dimitri A., Alsaraireh M.M., Alakash H., Al Yateem N., Zaghamir D.E., Elshatarat R.A., Subu M.A. (2024). Experiences on health-related quality of life of Jordanian patients living with heart failure: A qualitative study. PLoS ONE.

[4] Abu-Hantash H., Khader Y., Al-Saleh A., Al-Makhamreh H., Ibdah R., Ababneh M., Nammas A., Rasheed A., Toubasi A., Al-Qalalweh S. (2025). Clinical characteristics, management approaches, and outcomes of patients with heart failure: The Jordan Heart Failure Registry. Front. Cardiovasc. Med..

[5] Al-Balbissi K., Al-Saleh A., Al-Makhamreh H., Abu-Hantash H., Toubasi A., Albustanji F., Obaid Y.Y., Abu Tawileh H., Al-Qalalweh S., Mahmoud M.Y. (2025). Risk factors of mortality among heart failure patients in Jordan: The Jordanian Heart Failure Registry (JoHFR). Ann. Med. Surg..

[6] Moshki M., Khajavi A., Vakilian F., Minaee S., Hashemizadeh H. (2019). The content comparison of health-related quality of life measures in heart failure based on the international classification of functioning, disability, and health: A systematic review. J. Cardiovasc. Thorac. Res..

[7] Aburuz M.E. (2018). Anxiety and depression predicted quality of life among patients with heart failure. J. Multidiscip. Healthc..

[8] Comín-Colet J., Anguita M., Formiga F., Almenar L., Crespo-Leiro M.G., Manzano L., Muñiz J., Chaves J., De Frutos T., Enjuanes C. (2016). Health-related Quality of Life of Patients with Chronic Systolic Heart Failure in Spain: Results of the VIDA-IC Study. Rev. Esp. Cardiol. (Engl. Ed.).

[9] Jarab A.S., Hamam H.W., Al-Qerem W.A., Abu Heshmeh S.R., Mukattash T.L., Alefishat E.A. (2023). Health-related quality of life and its associated factors among outpatients with heart failure: A cross-sectional study. Health Qual. Life Outcomes.

[10] Ruku D.M., Chen H.M. (2024). The performance of physical activity and health-related quality of life in patients with heart failure: A cross-sectional study. J. Ners.

[11] Johansson I., Joseph P., Balasubramanian K., McMurray J.J.V., Lund L.H., Ezekowitz A.J., Kamath D., Alhabib K., Bayes-Genis A., Budaj A. (2021). Health-Related Quality of Life and Mortality in Heart Failure: The Global Congestive Heart Failure Study of 23,000 Patients from 40 Countries. Circulation.

[12] Moradi M., Daneshi F., Behzadmehr R., Rafiemanesh H., Bouya S., Raeisi M. (2020). Quality of life of chronic heart failure patients: A systematic review and meta-analysis. Heart Fail. Rev..

[13] Pequeno N.P.F., Cabral N.L.d.A., Marchioni D.M., Lima S.C.V.C., Lyra C.d.O. (2020). Quality of life assessment instruments for adults: A systematic review of population-based studies. Health Qual. Life Outcomes.

[14] Wilson I.B., Cleary P.D. (1995). Linking Clinical Variables with Health-Related Quality of Life: A Conceptual Model of Patient Outcomes. JAMA.

[15] Ferrans C.E., Zerwic J.J., Wilbur J.E., Larson J.L. (2005). Conceptual Model of Health-Related Quality of Life. J. Nurs. Scholarsh..

[16] Nave Leal E., Pais-Ribeiro J.L., Oliveira M.M., Silva M.N.D., Soares R., Fragata J., Ferreira R. (2010). Propriedades psicométricas da versão portuguesa do Kansas City Cardiomyopathy Questionnaire na miocardiopatia dilatada com insuficiência cardíaca congestiva. Rev. Port. Cardiol..

[17] Bilbao A., Escobar A., García-Perez L., Navarro G., Quirós R. (2016). The Minnesota living with heart failure questionnaire: Comparison of different factor structures. Health Qual. Life Outcomes.

[18] Green C., Porter C.B., Bresnahan D.R., Spertus J.A. (2000). Development and evaluation of the Kansas City Cardiomyopathy Questionnaire: A new health status measure for heart failure. J. Am. Coll. Cardiol..

[19] Wiklund I., Lindvall K., Swedberg K., Zupkis R.V. (1987). Self-assessment of quality of life in severe heart failure. An instrument for clinical use. Scand. J. Psychol..

[20] Guyatt G.H., Nogradi S., Halcrow S., Singer J., Sullivan M.J.J., Fallen E.L. (1989). Development and testing of a new measure of health status for clinical trials in heart failure. J. Gen. Intern. Med..

[21] de Louredo A.B., Leite A.L.C., Salerno G.R.F., Fernandes M., Blascovi-Assis S. (2015). Instruments to assess quality of life in patients with heart failure. Fisioter. Mov..

[22] Volterrani M., Halasz G., Adamopoulos S., Agostoni P.G., Butler J., Coats A.J.S., Cohen-Solal A., Doehner W., Filippatos G., Jankowska E. (2025). Quality of life in heart failure. The heart of the matter. A scientific statement of the Heart Failure Association and the European Association of Preventive Cardiology of the European Society of Cardiology. Eur. J. Prev. Cardiol..

[23] Dunderdale K., Thompson D.R., Miles J.N., Beer S.F., Furze G. (2005). Quality-of-life measurement in chronic heart failure: Do we take account of the patient perspective?. Eur. J. Heart Fail..

[24] Khajavi A., Moshki M., Minaee S., Vakilian F., Montazeri A., Hashemizadeh H. (2023). Chronic heart failure health-related quality of life questionnaire (CHFQOLQ-20): Development and psychometric properties. BMC Cardiovasc. Disord..

[25] Cameron J., Ski C.F., Thompson D.R. (2011). Cognitive impairment in chronic heart failure and the need for screening. Am. J. Cardiol..

[26] Bauer L.C., Johnson J.K., Pozehl B.J. (2011). Cognition in heart failure: An overview of the concepts and their measures. J. Am. Acad. Nurse Pract..

[27] Vogels R.L., Scheltens P., Schroeder-Tanka J.M., Weinstein H.C. (2007). Cognitive impairment in heart failure: A systematic review of the literature. Eur. J. Heart Fail..

[28] Brislin R.W. (1970). Back-Translation for Cross-Cultural Research. J. Cross Cult. Psychol..

[29] Wild D., Grove A., Martin M., Eremenco S., McElroy S., Verjee-Lorenz A., Erikson P. (2005). Principles of Good Practice for the Translation and Cultural Adaptation Process for Patient-Reported Outcomes (PRO) Measures: Report of the ISPOR Task Force for Translation and Cultural Adaptation. Value Health.

[30] Beaton D.E., Bombardier C., Guillemin F., Ferraz M.B. (2000). Guidelines for the process of cross-cultural adaptation of self-report measures. Spine.

[31] Juenger J., Schellberg D., Kraemer S., Haunstetter A., Zugck C., Herzog W., Haass M. (2002). Health related quality of life in patients with congestive heart failure: Comparison with other chronic diseases and relation to functional variables. Heart.

[32] Rector T.S., Cohn J.N. (1992). Assessment of patient outcome with the Minnesota Living with Heart Failure questionnaire: Reliability and validity during a randomized, double-blind, placebo-controlled trial of pimobendan. Pimobendan Multicenter Research Group. Am. Heart J..

[33] Tennant A., Conaghan P.G. (2007). The Rasch measurement model in rheumatology: What is it and why use it? When should it be applied, and what should one look for in a Rasch paper?. Arthritis Care Res..

[34] Li C.H. (2015). Confirmatory factor analysis with ordinal data: Comparing robust maximum likelihood and diagonally weighted least squares. Behav. Res. Methods.

[35] Andrich D. (1978). A Rating Formulation for Ordered Response Categories. Psychometrika.

[36] Lin C.-Y., Hammash M., Mudd-Martin G., Biddle M.J., Dignan M., Moser D.K. (2021). Older and Younger Patients’ Perceptions, Evaluations, and Responses to Worsening Heart Failure Symptoms. Heart Lung.

[37] Moser D.K., Heo S., Lee K.S., Hammash M., Riegel B., Lennie T.A., Arslanian-Engoren C., Mudd-Martin G., Albert N., Watkins J. (2013). “It Could Be Worse … Lot’s Worse!” Why Health-Related Quality of Life Is Better in Older Compared with Younger Individuals with Heart Failure. Age Ageing.

[38] Pandey A., Kitzman D., Reeves G. (2019). Frailty Is Intertwined with Heart Failure. JACC Heart Fail..

[39] Pastva A.M., Hugenschmidt C.E., Kitzman D.W., Nelson M.B., Brenes G.A., Reeves G.R., Mentz R.J., Whellan D.J., Chen H., Duncan P.W. (2021). Cognition, Physical Function, and Quality of Life in Older Patients with Acute Decompensated Heart Failure. J. Card. Fail..

[40] Lerdal A., Hofoss D., Gay C.L., Fagermoen M.S. (2019). Perception of Illness among Patients with Heart Failure Is Related to Their General Health Independently of Their Mood and Functional Capacity. J. Patient. Rep. Outcomes.

[41] Teresi J.A. (2006). Different Approaches to Differential Item Functioning in Health Applications. Advantages, Disadvantages and Some Neglected Topics. Med. Care.

[42] Schuler M., Musekamp G., Bengel J., Nolte S., Osborne R.H., Faller H. (2014). Measurement Invariance across Chronic Conditions: A Systematic Review and an Empirical Investigation of the Health Education Impact Questionnaire (HeiQ). Health Qual. Life Outcomes.

[43] Altman D.G., Royston P. (2006). The Cost of Dichotomising Continuous Variables. BMJ.

[44] Teresi J.A., Wang C., Kleinman M., Jones R.N., Weiss D.J. (2021). Differential Item Functioning Analyses of the Patient-Reported Outcomes Measurement Information System (PROMIS) Measures: Methods, Challenges, Advances, and Future Directions. Psychometrika.

[45] McMurray J.J.V., Pfeffer M.A. (2005). Heart failure. Lancet.

[46] Herr J.K., Salyer J., Flattery M., Goodloe L., Lyon D.E., Kabban C.S., Clement D.G. (2015). Heart failure symptom clusters and functional status—A cross-sectional study. J. Adv. Nurs..

[47] AbuRuz M.E., Alaloul F., Saifan A., Masa’DEh R., Abusalem S. (2015). Quality of Life for Saudi Patients with Heart Failure: A Cross-Sectional Correlational Study. Glob. J. Health Sci..

[48] Al-Sutari M.M., Abdalrahim M.S. (2024). Symptom Burden and Quality of Life Among Patients with Heart Failure. SAGE Open Nurs..

